# First molecular detection and genetic analysis of porcine circovirus 4 in the Southwest of China during 2021–2022

**DOI:** 10.3389/fmicb.2022.1052533

**Published:** 2022-11-03

**Authors:** Tong Xu, Dong You, Fang Wu, Ling Zhu, Xian-Gang Sun, Si-Yuan Lai, Yan-Ru Ai, Yuan-Cheng Zhou, Zhi-Wen Xu

**Affiliations:** ^1^College of Veterinary Medicine, Sichuan Agricultural University, Chengdu, China; ^2^College of Veterinary Medicine Sichuan Key Laboratory of Animal Epidemic Disease and Human Health, Sichuan Agricultural University, Chengdu, China; ^3^Animal Breeding and Genetics Key Laboratory of Sichuan Province, Sichuan Animal Science Academy, Chengdu, China; ^4^Livestock and Poultry Biological Products Key Laboratory of Sichuan Province, Sichuan Animal Science Academy, Chengdu, China

**Keywords:** porcine circovirus 4, the Southwest of China, molecular detection, genetic analysis, genotype

## Abstract

Porcine circovirus 4 (PCV4) was identified in 2019 as a novel circovirus species and then proved to be pathogenic to piglets. However, there is a lack of its prevalence in the Southwest of China. To investigate whether PCV4 DNA existed in the Southwest of China, 374 samples were collected from diseased pigs during 2021–2022 and detected by a real-time PCR assay. The results showed that the positive rate of PCV4 was 1.34% (5/374) at sample level, and PCV4 was detected in two of 12 cities, demonstrating that PCV4 could be detected in pig farms in the Southwest of China, but its prevalence was low. Furthermore, one PCV4 strain (SC-GA2022ABTC) was sequenced in this study and shared a high identity (98.1–99.7%) with reference strains at the genome level. Combining genetic evolution analysis with amino acid sequence analysis, three genotypes PCV4a, PCV4b, and PCV4c were temporarily identified, and the SC-GA2022ABTC strain belonged to PCV4c with a specific amino acid pattern (239V for Rep protein, 27N, 28R, and 212M for Cap protein). Phylogenetic tree and amino acid alignment showed that PCV4 had an ancient ancestor with mink circovirus. In conclusion, the present study was the first to report the discovery and the evolutionary analysis of the PCV4 genome in pig herds of the Southwest of China and provide insight into the molecular epidemiology of PCV4.

## Introduction

Porcine circoviruses (PCVs) are small, circular and single-strand DNA viruses belonging to the genus *Circovirus* of the family *Circoviridae* (Hamel et al., 1998; Meng, 2013; Opriessnig et al., 2020). At present, four species (PCV1, porcine circovirus 1; PCV2, porcine circovirus 2; PCV3, porcine circovirus 3; and PCV4, porcine circovirus 4) were recognized with similar structure. The genome contains two major open reading frames (ORFs): ORF1 and ORF2. The ORF1 gene encodes the replication-associated protein (Rep), and the ORF2 gene, in the opposite orientation from Rep, encodes the capsid protein (Cap) which was associated with the antigenic characteristics of circoviruses (Lekcharoensuk et al., 2004; Cheung, 2012).

Porcine circovirus 1 was first reported in 1974 and subsequently recognized non-pathogenic to pigs (Tischer et al., 1974, 1986; Allan et al., 1995), whereas, PCV2 has been deemed as one of the main agents of PCV-associated disease (PCVAD) (Nayar et al., 1997; Allan et al., 1998; Ellis et al., 1998; Kiupel et al., 1998; Morozov et al., 1998). PCVAD included post-weaning multisystem wasting syndrome (PMWS), porcine dermatitis and nephrotic syndrome (PDNS), and other syndromes (Nayar et al., 1997; Allan et al., 1998; Ellis et al., 1998; Kiupel et al., 1998; Morozov et al., 1998). In 2015, PCV3 was identified by next-generation sequencing analysis, and Jiang et al. (2019) have recently reported that PDNS-like disease can be reproduced in pigs infected with a cloned PCV3 virus (Phan et al., 2016; Palinski et al., 2017). In 2019, a novel circovirus species was identified in farmed pigs designated as PCV4, Hunan Province, China (Zhang D. et al., 2020), and later was also reported in several provinces and cities in China and South Korea (Chen et al., 2021; Sun et al., 2021a; Tian et al., 2021; Kim et al., 2022; Xu et al., 2022b). Recently, PCV4 was successfully rescued by Niu et al. (2022) from an infectious clone and demonstrated to be pathogenic to piglets. Nevertheless, genetic diversity and prevalence of PCV4 strains circulating in the Southwest of China have not been studied.

Therefore, in this study, a total of 374 samples were collected from diseased pigs with clinical signs of gastroenteritis (diarrhea) in pig farms in the Southwest of China and screened for the presence of PCV4 using a real-time PCR assay. Meanwhile, a complete genome sequence of PCV4 strain was generated and compared with published sequences in GenBank to study the genetic diversity of the PCV4 currently circulating in the Southwest of China during 2021–2022.

## Materials and methods

### Clinical samples collection and screening for porcine circovirus 4

A total of 374 clinical samples from pigs with clinical signs (PDNS, respiratory disease, and diarrhea) were collected from 37 farms in 12 cities (Mianyang, Suining, Chengdu, Deyang, Luzhou, Dazhou, Guangan, Guangyuan, Yibin, Nanchong, Neijiang, and Leshan) in the southwest of China from 2021 to 2022. The sample types in different farm comprised heart, liver, spleen, lung, kidney, brain, intestine, serum, nasal swab, and throat swab.

The viral genome was extracted using the FastPure Cell/Tissue DNA Isolation Mini Kit (Vazyme Biotech Co. Ltd., Nanjing, China) according to the manufacturer’s instructions. The RNA viral genome was extracted using Total RNA Kit I R6834 (Omega, Guangzhou, China) and then reverse-transcribed to cDNA using PrimeScript™ II 1st Strand cDNA Synthesis Kit (Takara, Dalian, China). DNA was detected for the presence of PCV4 using a SYBR Green I-based qPCR assay as described previously (Xu et al., 2022b). In addition, cDNA or DNA was also detected for eight viruses in pigs including porcine epidemic diarrhea virus (PEDV), porcine reproductive and respiratory syndrome virus (PRRSV), Porcine deltacoronavirus (PDCoV), swine acute diarrhea syndrome-coronavirus (SADS-CoV) pseudorabies virus (PRV), porcine circovirus 2 (PCV2), and porcine circovirus 3 (PCV3) using previously described PCR or qPCR assays (Kim et al., 2000; Wang et al., 2018; Zhao Y. et al., 2019; Zhou et al., 2019; Tian et al., 2020; Zheng et al., 2020).

### Complete genome sequencing of porcine circovirus 4

The complete genome of PCV4 was amplified as described previously (Xu et al., 2022a). The primers for amplifying the whole genome were summarized in Supplementary Table 1. The PCR reaction mixture consisted of 10 μl of PrimeSTAR^®^ Max DNA Polymerase (Takara, Beijing, China), 0.5 μl (25 μM) of forward and reverse primers, 1 μl of template DNA, and 8 μl of ddH_2_O. The thermistor parameters were as follows: initial incubation at 98°C for 3 min; 35 cycles of 20 s at 98°C, 60°C for 20 s, and 72°C for 45 s, and a final extension for 5 min at 72°C. The PCR products were purified using Gel Extraction Kit D2500 (Omega Bio-Tek) and then cloned into the pMD18-T Vector (Takara, Dalian, China) in accordance with the manufacturer’s instructions. Finally, three positive clones were sequenced directly by Sangon Biotech Shanghai Co. Ltd., China.

### Sequence alignment and phylogenetic analysis

The PCV4 whole genome was assembled using DNASTAR Lasergene. All unique PCV4 strains (Supplementary Table 2), available in GenBank database (accessed April 2, 2022) were analyzed with PCV4 strains in this study. To infer relationship of PCV4 and eight families of CRESS DNA viruses, *Rep* gene of 59 reference strains (Supplementary Table 2) that belong to eight families were downloaded from GenBank database. Molecular Evolutionary Genetics Analysis (MEGA) software (version 7.0) was used to the assemble, align, and analyze of the sequences. A neighbor-joining (NJ) phylogenetic tree was constructed with a p-distance model, and a bootstrap of 1,000 replicates using MEGA7.0.

## Results and discussion

In 2019, the first case of PCV4 infection was reported in Hunan Province, China. Since then, the genome of PCV4 has been identified in several provinces of China, including Jiangsu, Shanxi, Henan, Anhui, Guangxi, and Inner Mongolia (Zhang D. et al., 2020; Chen et al., 2021; Ha et al., 2021; Tian et al., 2021). Moreover, PCV4 DNA was also detected in Korea but not in Spain, Italy, and Colombia (Franzo et al., 2020; Nguyen et al., 2022; Vargas-Bermudez et al., 2022). Niu et al. (2022) have further identified that PCV4 was pathogenic to piglets. However, the prevalence and genetic evolution of PCV4 in the Southwest of China remains unclear. Therefore, extensive epidemiological studies of PCV4 in the Southwest of China should be conducted to better address the potential threat of this novel virus.

In this study, 374 samples from 37 pig farms located in 12 cities in the Southwest of China during 2021–2022 were screened to verify the presence of PCV4. The results showed that the positive rate of PCV4 was 1.34% (5/374), which was far lower than that of PCV4 (25.40%, 16/63) in pigs of Henan and Shanxi Provinces described by Tian et al. (2021). The prevalence of PCV4 at sample level was 1.32% (2/151) in 2021 and 1.34% (3/223) in 2022. These results suggested that PCV4 existed in the Southwest of China, but it is not widespread. In my opinion, it is necessary to take some measures to prevent and control the potential threat of PCV4. Two positive samples in 2021 were collected from newborn piglets from a farm suffered from severe watery and projectile diarrhea, and PEDV was positive in this farm (Table 1). Three positive samples in 2022 were collected from different growth stages from a pig farm suffered from PDNS, and PCV2 was also positive in this farm (Table 1). The pathogenicity of PCV4 infection alone warrants further study.

**TABLE 1:** Origin, clinical manifestation information and detection results of porcine circovirus 4 (PCV4) in pig clinical samples from the Southwest of China during 2021–2022.

Sample name	Collection date	Geographical location	Farm	Growth stages	Clinical symptoms	PEDV	TGEV	PDCoV	SADS	PRV	PCV2	PCV3	PRRSV
Sample 1	2021.11.02	Dazhou	Farm A	Newborn	Diarrhoea (3)	+	−	−	−	−	−	−	−
Sample 2	2021.11.02	Dazhou	Farm A	Newborn	Diarrhoea (3)	+	−	−	−	−	−	−	−
Sample 3※	2022.4.18	Guangan	Farm B	Weaning	PDNS	−	−	−	−	−	+	−	−
Sample 4	2022.4.18	Guangan	Farm B	Grower	PDNS	−	−	−	−	−	+	−	−
Sample 5	2022.4.18	Guangan	Farm B	Finisher	PDNS	−	−	−	−	−	+	−	−

The number in parentheses after the diarrhea symptom is the diarrhea score. The severity of diarrhea was scored based on clinical examination; 0, normal and no diarrhea; 1, mild and fluidic feces; 2, moderate watery diarrhea; 3, severe watery and projectile diarrhea; 4, death. The complete genome of SC-GA2022ABTC was acquired from sample 3 marked with ※.

**FIGURE 1:**
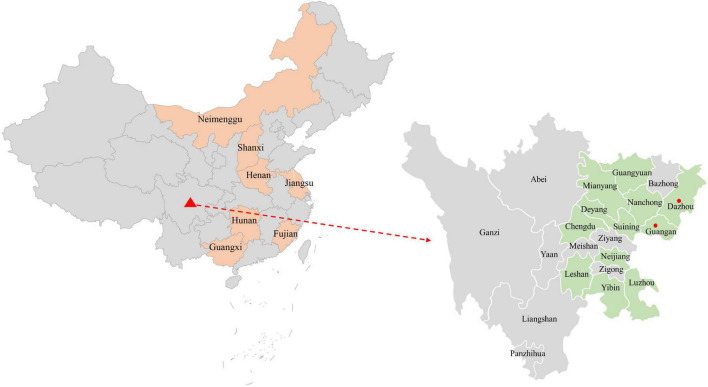
The geographical distribution of the 374 samples in the Southwest of China. In China, provinces with PCV4 complete genome amplified are filled with orange. In the Southwest of China, cities with sample collections are filled with light green, and cities with positive sample detection are marked with red solid circles.

The complete genome of one PCV4 strain (SC-GA2022ABTC) was amplified from a pig suffering from PDNS in Guangan, the Southwest of China, in 2022, and submitted to the GenBank database with the accession number OP497960. The complete genome of SC-GA2022ABTC was 1,770 nt in length without deletions and insertions of nucleotides. Compared with all 41 unique PCV4 strains (Supplementary Table 2) available in the GenBank database (accessed April 2, 2022), the SC-GA2022ABTC strain showed a high identity (98.1–99.8%) with PCV4 reference strains at the complete genome level. Among 41 PCV4 reference strains, SC-GA2022ABTC strain displayed the highest complete genome homology (99.8%) with HNU-AHG1-2019 derived from Hunan Province. As is depicted in Figure 1, among the provinces where the PCV4 whole genome has been reported, Hunan is the closest to Sichuan. Besides, 26 other representative circovirus strains (Supplementary Table 2) were selected for further analysis (Table 2). The genome of SC-GA2022ABTC strain showed the highest homology (68%) to the Mink circovirus, followed by 62.6% with Bat associated circovirus (accession no. NC 038385) and then to 35.8–51.6% with other circovirus species (Table 2), which was consistent with a previous report (Zhang H. H. et al., 2020). Additionally, SC-GA2022ABTC exhibited amino acid homology with these circovirus strains ranging from 16.2 to 80.1% for Rep proteins and 12.7 to 70.2% for Cap Proteins.

**TABLE 2:** Percent nucleotide and amino acid identity (% Identity) between porcine circovirus 4 (PCV4) strain in this study and reference strains.

				% identity				
				
Organism	Strain	Host	GenBank	Genome	Rep (nucleotide)	Rep (amino acid)	Cap (nucleotide)	Cap (amino acid)
Porcine circovirus 4	HNU-AHG1-2019	Pig	MK986820	99.7	99.7	99.3	99.7	99.6
Barbel circovirus	BaCV1	Barbus barbus	GU799606	41.9	50.6	46.8	31.3	27.1
Bat associated circovirus 1	XOR	Rhinolophus ferrumequinum	NC_038385	62.6	69.3	75.3	53.8	46
Bat circovirus	Acheng30	Vespertilio sinensis	NC_035799	43	56.9	51.9	36.1	23.9
Beak and feather disease virus	FJ-FZ01	Melopsittacus undulatus	MG148344	40.2	51	45.1	31.4	27
Canary circovirus	CCV	Serinus canaria	AJ301633	41.1	54.3	48.3	36.8	27.3
Canine circovirus	C85	Mongrel dog	MK944080	45.8	55.1	51.4	38.8	21.9
Chimpanzee stool avian-like circovirus	Chimp17	Chimpanzee	GQ404851	41.9	53.4	46	34.2	22.2
Columbid circovirus	coCV	Pigeon	AF252610	43	54.9	48.6	35.3	22.5
Cygnus olor circovirus	H51	Mute swan (Cygnus olor)	EU056309	42.7	52.8	48.8	32.1	26.5
Duck circovirus	FJZZ302	Duck	GQ423747	42.8	52.6	49.3	33.2	25.2
Finch circovirus	FiCV	Finch	DQ845075	43	55.2	48.8	35.7	28
Fox circovirus	55590	Vulpes vulpes	KP941114	46.6	56	51.7	37.2	21.4
Goose circovirus	JX1	Goose	GU320569	40.8	52	47	33	23.3
Gull circovirus	24	Lesser black-backed gull	KT454927	40.4	52.4	46.3	34.4	27.6
Human stool-associated circular virus	NG13	Homo sapiens	NC_038392	43.9	52.1	48.3	33.6	18.4
Mink circovirus	MiCV-DL13	Mink	NC_023885	68	73.3	80.1	61.4	70.2
Mulard duck circovirus	DuCV	Duck	AY228555	43.3	53.1	49.7	32.6	25.7
Porcine circovirus 1	PK	Pig	DQ650650	51.6	51.8	50.7	49.9	44.4
Porcine circovirus 2	TJ	Pig	AY181946	51.5	31.5	16.2	27.7	12.7
Porcine circovirus 3	FJ-PM01/2018	Pig	MK454951	42.9	53.2	48.4	33	24.8
Raven circovirus-4	4-1131	Corvus coronoides	DQ146997	41.6	52.4	47	37.4	25.3
Rhinolophus ferrumequinum circovirus 1	bat CV	Rhinolophus ferrumequinum	JQ814849	47.6	57.9	50	41.1	33.2
Silurus glanis circovirus	H5	Silurus glanis	JQ011377	41.4	49.6	48.8	32.6	21.6
Starling circovirus	StCV	European starling (Sturnus vulgaris)	DQ172906	42.4	53.7	49.8	32.7	24.1
Zebra finch circovirus	32469	Taeniopygia guttata (zebra finch)	KU641384	42.2	54.4	47	36.9	29.3

**FIGURE 2:**
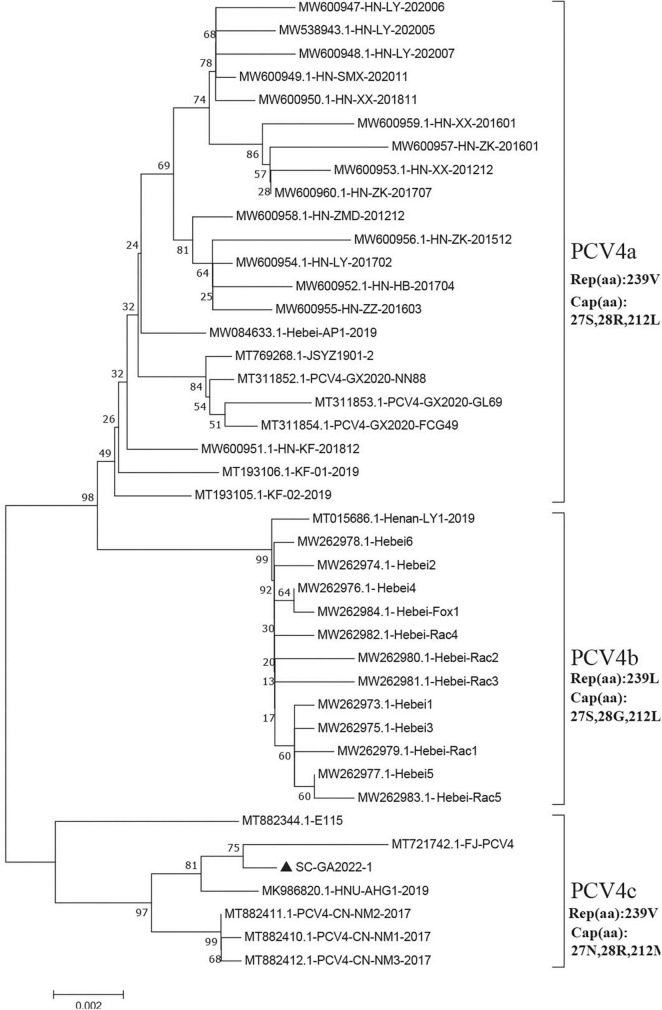
Neighbor-joining trees were constructed with a p-distance model and bootstrapping at 1,000 replicates. Phylogenetic tree was constructed based on the complete genome of 42 PCV4 strains. SC-GA2022ABTC strain was marked with the black solid triangle (▲). Scale bar indicates nucleotide substitutions per site.

To address the evolutionary relationship between the SC-GA2022ABTC strain and PCV4 strains in other regions, a phylogenetic tree was constructed based on the complete genome sequences of the SC-GA2022ABTC strain and 41 reference strains, and PCV4 was temporarily divided into three genotypes (PCV4a, PCV4b, and PCV4c) (Figure 2; Xu et al., 2022a). A total of 22 PCV4 strains from four provinces (Henan, Hebei, Guangxi, and Jiangsu) of China belonged to PCV4a; 13 reference strains derived from two provinces (Henan and Hebei) belonged to PCV4b. Nevertheless, the SC-GA2022ABTC strain and six PCV4 strains from three provinces (Fujian, Hunan, and Inner Mongolia) of China were clustered into PCV4c together with one South Korea strain.

Moreover, four amino acid mutations (V239L in Rep, S27N, R28G, and L212M in Cap) could serve as a molecular marker for PCV4 clade divisions (Xu et al., 2022a), which was also observed in this study (Figure 2). Summarizing the marker codons in Rep and Cap resulted in a specific amino acid pattern for PCV4a (239V in Rep, 27S, 28R, and 212L in Cap), for PCV4b (239L in Rep, 27S, 28G, and 212L in Cap) and for PCV4c (239V for Rep protein, 27N, 28R, and 212M for Cap protein). Amino acid substitutions as markers for clade divisions of other viruses were also reported as described previously, such as PCV3 and CPV (Parrish et al., 1988, 1991; Zhao et al., 2016; Fu et al., 2018; Kwan et al., 2021). Compared with previous studies (Hou et al., 2021; Xu et al., 2022b), more whole-genome sequences were available in the Genbank database, and the genetic tree was also richer, which could aid an easier interpretation of PCV-4 molecular epidemiology. Therefore, greater efforts must be made to provide more representative and structured sampling campaigns and to increase the sharing of correctly annotated sequences in free databases, in order to establish more accurate and scientific typing schemes.

**FIGURE 3:**
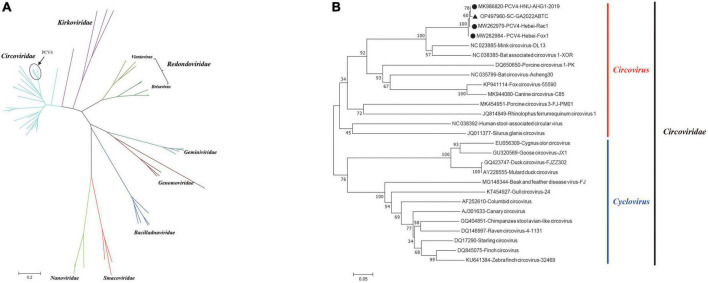
Neighbor-joining trees were constructed with a p-distance model and bootstrapping at 1,000 replicates. **(A)** Phylogenetic tree was constructed based on amino acids of *Rep* of 63 *CRESS* DNA virus strains. **(B)** Phylogenetic tree was constructed based on amino acids of *Rep* of 27 *Circoviridae* strains. SC-GA2022ABTC strain was marked with the black solid triangle (▲). The other three Porcine circovirus 4 (PCV4) reference strains were marked with black filled circles (⬤). Scale bar indicates nucleotide substitutions per site.

The circular Rep-encoding single stranded (CRESS) DNA virus emergence in diverse host has been associated with severe diseases (Zhao L. et al., 2019). Seven family members of the CRESS DNA viruses have been reported, including *Circovidae, Nanoviridae, Smacoviridae, Genomoviridae, Bacilladnaviridae, Geminiviridae*, and *Redondoviridae*, established by the International Committee on the Taxonomy of Viruses (ICTV). Recently, a proposed family Kirkoviridae emerged and was reported in several recent studies (Liu et al., 2021; Sun et al., 2021b). Moreover, porcine circovirus-like virus P1 is an unclassified circovirus and might be one of the agents of PCVAD, while its replication-related proteins remained unknown (Wen et al., 2018). To better understand the evolutionary origin of PCV4, a data set including the PCV4 strain in our study, three PCV4 reference strain and 59 other representative CRESS DNA virus strains was analyzed. Then, a phylogenetic tree was constructed based on the amino acids of *Rep* to explore the origin of PCV 4 (Figure 3A). As is depicted in Figure 3A, four PCV4 strains (including SC-GA2022ABTC) were clustered into *Circovidae*. The genus *Redondoviridae* includes the species *Brisavirus* and *Vientovirus* (Figure 3A), and *Circovidae* two genera (*Cyclovirus* and *Circovirus*) (Figure 3B). The phylogenetic tree based on the amino acids of Rep of *Circovidae* strains showed that PCV4 was distantly related to other PCVs but closely related to mink circovirus (Figure 3B), which was corroborated by complete genome homology of PCV4 strain in this study with reference strains (Table 1). These results suggested that PCV4 shares an ancient ancestor with mink circovirus, which was consistent with a previous study (Li et al., 2022).

**FIGURE 4:**
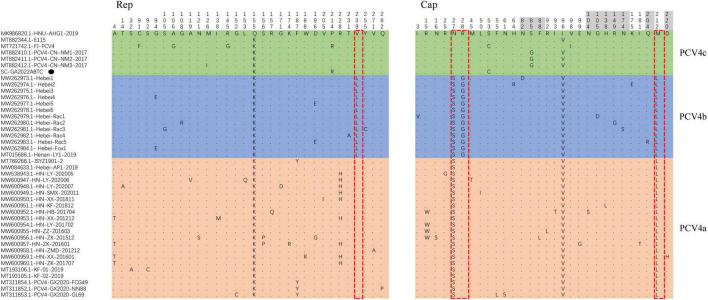
All amino acid mutation sites of Rep protein and Cap of 42 Porcine circovirus 4 (PCV4) strains. PCV4 strains were divided into three genotypes, comprising PCV4a (light orange), PCV4b (light green), PCV4c (light blue). The specific amino acid patterns of genotype were displayed in the red dotted box. Amino acid residues contained in potential linear B-cell epitopes are highlighted in light grey. SC-GA2022ABTC strain investigated in this study. SC-GA2022ABTC strain sequenced in this study was marked with the black solid triangle (▲).

Among 42 PCV4 strains, 32 amino acid mutations were observed in the Rep (Figure 4). For Rep, the N-terminal endonuclease domain comprised three conserved motifs (motifI-^13^FTLNN^17^, motifII-^50^PHLQG^54^, and motifIII-^90^YCSK^93^) and the helicase domain of superfamily 3 (SF3) containing three Walker motifs (Walker A-^168^GxxxxGKS^175^, Walker B-^207^DDY^209^, and Walker C-^245^ITSN^248^) were observed in SC-GA2022ABTC strain, which was consistent with a previous study (Nguyen et al., 2022).

For the Caps of PCVs (PCV1–PCV3), the nuclear localization signal (NLS) region, an arginine-rich region within the circovirus genus, mediate nuclear targeting of viral genomes and was experimentally confirmed (Liu et al., 2001; Shuai et al., 2008; Mou et al., 2019), which was also confirmed in the N-terminus of PCV4 Cap that ranged from 1 to 20 amino acid (Zhou et al., 2021). Compared to other PCV4 reference strains, no amino acid mutations occurred in the NLS region of the SC-GA2022ABTC strain. However, the amino acid mutations (I3V for Hebei-Rac1 strain, R15W for HN-HB-201704 strain, HN-LY-201702 strain, HN-ZZ-201603 strain, and HN-ZK-201512 strain, and N16S for HN-ZK-201512) were different from other PCV4 strains and occurred in the NLS region, indicating these PCV4 strains might differ in cell tropism, manner and speed of cell entry. Five potential linear B-cell epitopes with high antigenicity were predicted in a recent report, including Epitope A: 72F-88F; Epitope B: 104N-112Y; Epitope C: 122D-177N; Epitope D: 199N-205V; and Epitope E: 219F-225P (Wang et al., 2021). As shown in Figure 4, there were 30 amino acid mutations in Cap of 42 PCV4 strains, 11 of which were located in the predicted epitope region, which may alter the antigenicity of the PCV4 Cap. However, potential immunogenic changes due to amino acid mutations in epitope regions of Cap remained to be determined.

## Conclusion

Overall, this study was the first to report the presence of PCV4 in the Southwest of China. The first complete genome sequence in the Southwest of China was successfully sequenced and named SC-GA2022ABTC strain. The SC-GA2022ABTC strain shared a high homology (98.1–99.7%) with other PCV4 strains. SC-GA2022ABTC strain belonged to PCV4c with a specific amino acid pattern (239V for Rep protein, 27N, 28R, and 212M for Cap protein) by combining genetic evolution with amino acid sequence. These findings help us to understand the prevalence and genomic characteristics of PCV4 in pig farms in the Southwest of China.

## Data availability statement

The data presented in this study are deposited in the GenBank repository, accession number: OP497960.

## Ethics statement

All experimental procedures were reviewed and approved by the Sichuan Agricultural University Animal Care and Use Committee [license number SCXK (Sichuan) 2013-0001].

## Author contributions

X-GS, S-YL, and LZ contributed to the conceptualization. Y-CZ and Y-RA contributed to the methodology. TX contributed to the software and writing—original draft preparation. FW and DY contributed to the validation and supervision. Z-WX contributed to the project administration and funding acquisition. All authors contributed to the article and approved the submitted version.
