# Supplementary material for: First molecular detection and genetic analysis of porcine circovirus 4 in the Southwest of China during 2021–2022
Source: Front Microbiol. 2022 Nov 3;13:1052533. doi: 10.3389/fmicb.2022.1052533 (PMC9668871; doi:10.3389/fmicb.2022.1052533)
Supplement: Supplementary file 1 [file Data_Sheet_1.docx]

Table S1 List of primer sequences used in this study

| Primer name | Nucleotide sequence (5’-3’) | Primer locations (bp) | Product size | |
| --- | --- | --- | --- | --- |
| PCV4-1F | GAGGTTCCACCCGTTTAAG | 260-278 | 577 |  |
| PCV4-1R | CCAGTCCTTGATCTGCTTGTTG | 815-836 |  |  |
| PCV4-2F | GCCAAGACAATGTGGATTACC | 792-812 | 690 |  |
| PCV4-2R | AGCCTCCCATTTGCATATTACC | 1460-1481 |  |  |
| PCV4-3F | CCACATAGTCTCCATCCAGTTG | 1361-1382 | 769 |  |
| PCV4-3R | CCCTCCTTTGGAGCAATACTT | 339-359 |  |  |

Table S2 The information of reference strains for sequence alignment and phylogenetic analysis

| Strain | Organism | Size | Collection date | Country | Accession number | Family | Strain | Organism | Size | Collection date | Country | Accession number | Family |
| --- | --- | --- | --- | --- | --- | --- | --- | --- | --- | --- | --- | --- | --- |
| HNU-AHG1-2019 | Porcine circovirus 4 | 1770 | Feb-2019 | China | MK986820.1 | *Circoviridae* | HN-ZK-201707 | Porcine circovirus 4 | 1770 | Jul-2017 | China | MW600960.1 | *Circoviridae* |
| Henan-LY1-2019 | Porcine circovirus 4 | 1770 | Feb-2019 | China | MT015686.1 | *Circoviridae* | BaCV1 | Barbel circovirus | 1957 | 02-Jul-2008 | Hungary | GU799606 | *Circoviridae* |
| KF-02-2019 | Porcine circovirus 4 | 1770 | Oct-2019 | China | MT193105.1 | *Circoviridae* | XOR | Bat associated circovirus 1 | 1862 | Nov-2008 | Myanmar | NC_038385 | *Circoviridae* |
| KF-01-2019 | Porcine circovirus 4 | 1770 | Oct-2019 | China | MT193106.1 | *Circoviridae* | Acheng30 | Bat circovirus | 2113 | 2016 | China | NC_035799 | *Circoviridae* |
| PCV4/GX2020/NN88 | Porcine circovirus 4 | 1770 | 2018 | China | MT311852.1 | *Circoviridae* | FJ-FZ01 | Beak and feather disease virus | 2003 | May-2016 | China | MG148344 | *Circoviridae* |
| PCV4/GX2020/GL69 | Porcine circovirus 4 | 1770 | 2018 | China | MT311853.1 | *Circoviridae* | CCV | Canary circovirus | 1952 | 2003 | United Kingdom | AJ301633 | *Circoviridae* |
| PCV4/GX2020/FCG49 | Porcine circovirus 4 | 1770 | 2018 | China | MT311854.1 | *Circoviridae* | C85 | Canine circovirus | 2063 | Apr-2016 | China | MK944080 | *Circoviridae* |
| FJ-PCV4 | Porcine circovirus 4 | 1770 | 2019 | China | MT721742.1 | *Circoviridae* | Chimp17 | Chimpanzee stool avian-like circovirus | 1935 | Sep-2002 | Rwanda | GQ404851 | *Circoviridae* |
| JSYZ1901-2 | Porcine circovirus 4 | 1770 | 02-Jan-2019 | China | MT769268.1 | *Circoviridae* | coCV | Columbid circovirus | 2037 | 2001 | Germany | AF252610 | *Circoviridae* |
| E115 | Porcine circovirus 4 | 1770 | 23-Apr-2020 | South Korea | MT882344.1 | *Circoviridae* | H51 | Swan circovirus | 1783 | 2006 | Germany | EU056309 | *Circoviridae* |
| PCV4/CN/NM1/2017 | Porcine circovirus 4 | 1770 | 2017 | China | MT882410.1 | *Circoviridae* | FJZZ302 | Duck circovirus | 1995 | 15-Dec-2008 | China: | GQ423747 | *Circoviridae* |
| PCV4/CN/NM2/2017 | Porcine circovirus 4 | 1770 | 2017 | China | MT882411.1 | *Circoviridae* | FiCV | Finch circovirus | 1962 | 2007 | United Kingdom | DQ845075 | *Circoviridae* |
| PCV4/CN/NM3/2017 | Porcine circovirus 4 | 1770 | 2017 | China | MT882412.1 | *Circoviridae* | 55590 | Fox circovirus | 2055 | 2014 | Croatia | KP941114 | *Circoviridae* |
| Hebei-AP1-2019 | Porcine circovirus 4 | 1770 | 2019 | China | MW084633.1 | *Circoviridae* | JX1 | Goose circovirus | 1821 | Oct-2009 | China: Jiangxi | GU320569 | *Circoviridae* |
| Hebei1 | Porcine circovirus 4 | 1770 | 10-Sep-2020 | China | MW262973.1 | *Circoviridae* | 24 | Gull circovirus | 2035 | 20-Aug-2014 | Netherlands | KT454927 | *Circoviridae* |
| Hebei2 | Porcine circovirus 4 | 1770 | 15-Sep-2020 | China | MW262974.1 | *Circoviridae* | UN | Gull circovirus | 2035 | 2009 | Germany | JQ685854 | *Circoviridae* |
| Hebei3 | Porcine circovirus 4 | 1770 | 15-Sep-2020 | China | MW262975.1 | *Circoviridae* | NG13 | Human stool-associated circular virus | 1699 | 2007 | Nigeria | NC_038392 | *Circoviridae* |
| Hebei4 | Porcine circovirus 4 | 1770 | 20-Sep-2020 | China | MW262976.1 | *Circoviridae* | MiCV-DL13 | Mink circovirus | 1753 | 30-Oct-2013 | China | NC_023885 | *Circoviridae* |
| Hebei5 | Porcine circovirus 4 | 1770 | 20-Sep-2020 | China | MW262977.1 | *Circoviridae* | DuCV | Mulard duck circovirus | 1996 | 2003 | Germany | AY228555 | *Circoviridae* |
| Hebei6 | Porcine circovirus 4 | 1770 | 20-Sep-2020 | China | MW262978.1 | *Circoviridae* | PK | Porcine circovirus 1 | 1759 | 2006 | China | DQ650650. | *Circoviridae* |
| Hebei-Rac1 | Porcine circovirus 4 | 1770 | 01-Oct-2015 | China | MW262979.1 | *Circoviridae* | TJ | Porcine circovirus 2 | 1767 | 2009 | China | AY181946 | *Circoviridae* |
| Hebei-Rac2 | Porcine circovirus 4 | 1770 | 07-Nov-2017 | China | MW262980.1 | *Circoviridae* | FJ-PM01/2018 | Porcine circovirus 3 | 2000 | Sep-2018 | China | MK454951 | *Circoviridae* |
| Hebei-Rac3 | Porcine circovirus 4 | 1770 | 16-Jun-2019 | China | MW262981.1 | *Circoviridae* | 4-1131 | Raven circovirus | 1898 | 2005 | Australia | DQ146997 | *Circoviridae* |
| Hebei-Rac4 | Porcine circovirus 4 | 1770 | 13-Jun-2018 | China | MW262982.1 | *Circoviridae* | bat CV | Rhinolophus ferrumequinum circovirus 1 | 1760 | Feb-2011 | China | JQ814849.1 | *Circoviridae* |
| Hebei-Rac5 | Porcine circovirus 4 | 1770 | 02-Jun-2018 | China | MW262983.1 | *Circoviridae* | H5 | Silurus glanis circovirus | 1966 | 26-Sep-2011 | Hungary | JQ011377.1 | *Circoviridae* |
| Hebei-Fox1 | Porcine circovirus 4 | 1770 | 25-Jun-2018 | China | MW262984.1 | *Circoviridae* | UN | Starling circovirus | 2063 | 2005 | Germany | DQ172906 | *Circoviridae* |
| HN-LY-202005 | Porcine circovirus 4 | 1770 | May-2020 | China | MW538943.1 | *Circoviridae* | 32469 | Zebra finch circovirus | 1983 | 2014 | Germany | KU641384. | *Circoviridae* |
| HN-LY-202006 | Porcine circovirus 4 | 1770 | Jun-2020 | China | MW600947.1 | *Circoviridae* | SS10-8V | Chaetoceros tenuissimus DNA virus type-II | 5770 | 2010 | Japan | AB971658 | *Bacilladnaviridae* |
| HN-LY-202007 | Porcine circovirus 4 | 1770 | Jul-2020 | China | MW600948.1 | *Circoviridae* | LDMD-2013 | Bacillariodnavirus | 5749 | 03-Mar-2009 | USA | KF133809 | *Bacilladnaviridae* |
| HN-SMX-202011 | Porcine circovirus 4 | 1770 | Nov-2020 | China | MW600949.1 | *Circoviridae* | GaBacV1 | Amphibola crenata associated bacilladnavirus 1 | 4729 | 2012 | New Zealand | KY405006 | *Bacilladnaviridae* |
| HN-XX-201811 | Porcine circovirus 4 | 1770 | Nov-2018 | China | MW600950.1 | *Circoviridae* | GaBacV2 | Amphibola crenata associated bacilladnavirus 2 | 4576 | 2012 | New Zealand | KY405007 | *Bacilladnaviridae* |
| HN-KF-201812 | Porcine circovirus 4 | 1770 | Dec-2018 | China | MW600951.1 | *Circoviridae* | CtenDNAV06 | Chaetoceros protobacilladnavirus 4 | 5639 | 2005 | Japan | AB597949 | *Bacilladnaviridae* |
| HN-HB-201704 | Porcine circovirus 4 | 1770 | Apr-2017 | China | MW600952.1 | *Circoviridae* | Bean | Macroptilium mosaic Puerto Rico virus | 2615 | 2003 | Puerto Rico | AF449192 | *Geminiviridae* |
| HN-XX-201212 | Porcine circovirus 4 | 1770 | Dec-2012 | China | MW600953.1 | *Circoviridae* | DNA-A | Corchorus golden mosaic virus | 2677 | 2008 | Viet Nam | DQ641688 | *Geminiviridae* |
| HN-LY-201702 | Porcine circovirus 4 | 1770 | Feb-2017 | China | MW600954.1 | *Circoviridae* | 28:2007 | Rhynchosia golden mosaic Havana | 2609 | 2007 | Cuba | HM236368 | *Geminiviridae* |
| HN-ZZ-201603 | Porcine circovirus 4 | 1770 | Mar-2016 | China | MW600955.1 | *Circoviridae* | Nsukka | Cowpea golden mosaic viru | 2728 | 1990 | Nigeria | AF029217 | *Geminiviridae* |
| HN-ZK-201512 | Porcine circovirus 4 | 1770 | Dec-2015 | China | MW600956.1 | *Circoviridae* | BS3913 | Sewage-associated gemycircularvirus 4 | 2115 | 2015 | New Zealand | KJ547634 | *Genomoviridae* |
| HN-ZK-201601 | Porcine circovirus 4 | 1770 | Jan-2016 | China | MW600957.1 | *Circoviridae* | BS4014 | Sewage-associated gemycircularvirus 6 | 2147 | 2012 | New Zealand | KJ547636 | *Genomoviridae* |
| HN-ZMD-201212 | Porcine circovirus 4 | 1770 | Dec-2012 | China | MW600958.1 | *Circoviridae* | 52_Fec78023_cow | Faeces associated gemycircularvirus 2 | 2164 | 2012 | New Zealand | KT862253 | *Genomoviridae* |
| HN-XX-201601 | Porcine circovirus 4 | 1770 | Jan-2016 | China | MW600959.1 | *Circoviridae* |  | Sclerotinia sclerotiorum hypovirulence | 2166 | 2010 | China | GQ365709 | *Genomoviridae* |

Note: UN, Unknow.

Table S2 (Continued)

| Strain | Organism | Size | Collection date | Country | Accession number | Family | Strain | Organism | Size | Collection date | Country | Accession number | Family |  |
| --- | --- | --- | --- | --- | --- | --- | --- | --- | --- | --- | --- | --- | --- | --- |
| TOS28 | Banana bunchy top viru | 1109 | 2010 | Tonga | JF957636 | *Nanoviridae* | DC | Human lung-associated vientovirus | 3053 | 2018 | UN | MK059761 | *Redondoviridae* | GX14 |
| 548_BI_1995 | Banana bunchy top virus | 1111 | 2011 | Burundi | KM607601 | *Nanoviridae* | MC | Human oral-associated vientovirus | 3058 | 2018 | UN | MK059770 | *Redondoviridae* |  |
| JKI-1998/99 | Faba bean necrotic stunt virus | 1003 | 2010 | Ethiopia | AJ749894 | *Nanoviridae* | 15040 | Human respiratory circular DNA virus | 3054 | 2015 | Spain | KY244146 | *Redondoviridae* |  |
| Eth-231 | Faba bean yellow leaf virus | 1002 | 2013 | Ethiopia | HE654123 | *Nanoviridae* | 15037 | Human respiratory circular DNA viru | 3054 | 2014 | Spain | KY328745 | *Redondoviridae* |  |
| MD | Human lung-associated brisavirus | 3050 | 2019 | USA | MK059756 | *Redondoviridae* | DcSCV_c1358 | Camel associated porprismacovirus 4 | 2575 | 2013 | United Arab Emirates | KM573775 | *Smacoviridae* |  |
| RC | Human lung-associated brisavirus | 3026 | 2019 | USA | MK059757 | *Redondoviridae* | 47_Fec60415_sheep | Sheep faeces associated smacovirus 2 | 2712 | 2012 | New Zealand | KT862221 | *Smacoviridae* |  |
| YH | Human oral-associated brisavirus | 3034 | 2019 | USA | MK059758 | *Redondoviridae* | DcSCV_c1378 | Camel associated porprismacovirus 1 | 2565 | 2013 | United Arab Emirates | KM573772 | *Smacoviridae* |  |
| VW | Human gut-associated brisavirus | 3030 | 2019 | USA | MK059759 | *Redondoviridae* | GX14 | Po-Circo-like virus isolate | 3944 | 2018 | China | MN263296 | *Kirkoviridae* |  |
| MRJ | Human PoSCV5-like circular virus | 3018 | 2016 | China | KY052047 | *Redondoviridae* | cg4648 | Circular ssDNA virus sp | 290 aa | 2014 | USA | APG55803※ | *Kirkoviridae* |  |
| 15278 | Circoviridae sp. | 3019 | 2016 | Spain | KY349925 | *Redondoviridae* | Tarto | Human fecal virus Tarto | 307 aa |  | Estoni | ASH99030※ | *Kirkoviridae* |  |
| UN | Human lung-associated brisavirus II | 3023 | 2018 | UN | MK059755 | *Redondoviridae* | CH | Bo-Circo-like virus | 307 aa | 2016 | China | AXK90322.1※ | *Kirkoviridae* |  |
| 15232 | Human respiratory circular DNA virus | 3054 | 2015 | Spain | KY328746 | *Redondoviridae* |  |  |  |  |  |  |  |  |

Note: three accession numbers (APG55803, ASH99030 and AXK90322) labeled with※ were the amino acid sequences of Rep, and their “size” represents the length of the amino acid. Other accession numbers represent nucleotide sequences.
